# News

**DOI:** 10.1155/S1463924601000050

**Published:** 2001


### News

### Preconceived ideas for chemical industry

*London, 16 June 2000:* A preconceived business package solution for middle-sized companies in the chemical industry has been launched in London.

*myCHEM@icm* is a quickly installed SAP-certified business package that is bound to have appeal for companies that have up to now avoided the implementation of costly software.

ICM UK's Managing Director, Adrian Bray said: 'With this certification, ICM has succeeded in widening the gap with its competitors, since it is the first consulting agency worldwide that has developed a business package solution certified by SAP for the chemical industry'.

The previous week at ACHEMA in Frankfurt, Siegfried Kalugin, In-house Systems Manager for SAP AG, handed to the ICM Group the certified approval document for myCHEM@icm.

According to Kalugin: 'partners like the Systems Company ICM, who can offer high-quality products, are very important for SAP AG when it comes to the middle-sized business arena'.

Already included in its functional capacity are maintenance and support, training manuals, user documentation, process and function descriptions, training for the key user as well as special tools for the adoption of old data.

As a result of several years' experience in the chemical industry, ICM has succeeded in getting this solution certified by SAP AG within only four months.

Over the next few months the ICM Group GmbH will also be awarded certifications for their business packaged solutions *myFOOD@icm* for the food industry and myPHARMA@icm for the pharmaceutical industry.

*For further information please contact: Adrian Bray at ICM Management Consulting Limited. Tel: + 44 (0)208 334 8032; e-mail: Abray@icm.de*

### Delivery of Allegro™ UHTS Systems

*Hopkinton, MA—December 2000:* Zymark Corporation announces the recent delivery of three Allegro™ UHTS Systems to Pfizer Global Research and Development. The Ann Arbor, Michigan, facility will use two of the systems, while the other will be located in Pfizer's San Diego, California, laboratory. Allegro is Zymark's celebrated automated screening system with the unique industrial assembly-line approach of individual modular workstations that received the distinguished 1999 R&D 100 Innovative Product Award for outstanding technological advances.

'We are thrilled about the advances in drug discovery Pfizer has the potential to make utilizing our Allegro system', said Kevin Hrusovsky, Zymark Corporation President and CEO. 'It is another major milestone for Zymark to have three Allegro installations actively screening and be associated with such a first class team. Their high level of commitment and enthusiasm for these technologies provides them with a major advantage in the race to identify promising new chemical leads.'

Zymark's Allegro automation technology is designed specifically to be a high performance tool for drug discovery, and is constructed with modular workstation units to meet the intense flexibility requirements of drug discovery research. The system has developed a reputation for operating reliably under the most demanding of circumstances, and new 384 and 1536 dispensing technology will increase Allegro's screening throughput to over 400 000 assay points per day. Allegro is compatible with virtually all conventional assay techniques, and the reconfigurable system's open architecture ensures forward compatibility with advances in assay chemistry.

Zymark Corporation impacts the world by advancing the ability to discover, develop, and market safe and effective drugs that can lengthen and improve the quality of life for everyone. Zymark's employees are active in more than 35 countries and their combined expertise in Pharmaceutical Analysis and Laboratory Automation makes Zymark the world's leading supplier in this application area.

*For additional information contact Jordana Estner, Marketing Communications. Tel: + 1 (508) 497-6541, e-mail: Jordana.Estner@zymark.com*

*Journal of Automated Methods & Management in Chemistry* ISSN 1463–9246 print/ISSN 1464–5068 online © 200? Taylor & Francis Ltd
http://www.tandf.co.uk/journals
DOI: 10.1080/146392401143787

